# The earth’s gravity field recovery using the third invariant of the gravity gradient tensor from GOCE

**DOI:** 10.1038/s41598-021-81840-1

**Published:** 2021-02-11

**Authors:** Lin Cai, Xiaoyun Wan, Houtse Hsu, Jiangjun Ran, Xiangchao Meng, Zhicai Luo, Zebing Zhou

**Affiliations:** 1 MOE Key Laboratory of Fundamental Physical Quantities Measurement, Hubei Key Laboratory of Gravitation and Quantum Physics, School of Physics, Huazhong University of Science and Technology, Wuhan, 430074 China; 2 Institute of Geophysics, Huazhong University of Science and Technology, Wuhan, 430074 China; 3 School of Land Science and Technology, China University of Geosciences (Beijing), Beijing, 100083 China; 4 Institute of Geodesy and Geophysics (IGG), Chinese Academy of Sciences, Wuhan, 430077 China; 5 Department of Earth and Space Sciences, Southern University of Science and Technology, Shenzhen, 518055 China; 6 First Crust Deformation Monitoring and Application Center, China Earthquake Administration, Tianjin, 300180 China

**Keywords:** Geodynamics, Geophysics

## Abstract

Due to the independence of the gradiometer instrument’s orientation in space, the second invariant $I_2$ of gravity gradients in combination with individual gravity gradients are demonstrated to be valid for gravity field determination. In this contribution, we develop a novel gravity field model named I3GG, which is built mainly based on three novel elements: (1) proposing to utilize the third invariant $I_3$ of the gravity field and steady-state ocean circulation explorer (GOCE) gravity gradient tensor, instead of using the $I_2$, similar to the previous studies; (2) applying an alternative two-dimensional fast fourier transform (2D FFT) method; (3) showing the advantages of $I_3$ over $I_2$ in the effect of measurement noise from the theoretical and practical computations. For the purpose of implementing the linearization of the third invariant, this study employs the theory of boundary value problems with sphere approximation at an accuracy level of $O(J_2^2\cdot T_{ij})$. In order to efficiently solve the boundary value problems, we proposed an alternative method of 2D FFT, which uses the coherent sampling theory to obtain the relationship between the 2D FFT and the third invariant measurements and uses the pseudo-inverse via QR factorization to transform the 2D Fourier coefficients to spherical harmonic ones. Based on the GOCE gravity gradient data of the nominal mission phase, a novel global gravity field model (I3GG) is derived up to maximum degree/order 240, corresponding to a spatial resolution of 83 km at the equator. Moreover, in order to investigate the differences of gravity field determination between $I_3$ with $I_2$, we applied the same processing strategy on the second invariant measurements of the GOCE mission and we obtained another gravity field model (I2GG) with a maximum degree of 220, which is 20 degrees lower than that of I3GG. The root-mean-square (RMS) values of geoid differences indicates that the effects of measurement noise of I3GG is about 20% lower than that on I2GG when compared to the gravity field model EGM2008 (Earth Gravitational Model 2008) or EIGEN-5C (EIGEN: European Improved Gravity model of the Earth by New techniques). Then the accuracy of I3GG is evaluated independently by comparison the RMS differences between Global Navigation Satellite System (GNSS)/leveling data and the model-derived geoid heights. Meanwhile, the re-calibrated GOCE data released in 2018 is also dealt with and the corresponding result also shows the similar characteristics.

## Introduction

The GOCE (Gravity Field and Steady-state Ocean Circulation Explorer) satellite is the first mission to apply the principle of the satellite gravity gradiometry in space to provide the Earth’s gravity field models on a global scale with high spatial resolution and very high accuracy^1^. For this purpose, GOCE is equipped with a sensitive electrostatic gravity gradiometer (EGG), which consists of six electrostatic accelerometers and measures the gradient tensor of the Earth’s gravity field^2,3^. Much effort has been made to recover the Earth’s gravity field from the gravity gradients data collected by GOCE, especially for the main diagonal elements of the gravitational tensor^4–6^. It is known that the gravity gradients are linearly linked with the gravity potential coefficients, which are usually solved by the space-wise or time-wise approaches^7,8^. However, when dealing with the gravity data of GOCE, it is problematic when transforming the gravity gradients from the gradiometer reference frame (GRF) to the other reference frames by applying the star camera data. The error in the transformation among different coordinate systems is investigated by the studies^9–11^, which demonstrate that the GOCE gradiometer orientation, due to errors in the reconstitution procedure of the satellite’s attitude, becomes one of the main error sources in the GOCE data processing, if the root mean square (RMS) of attitude errors exceeds 3–5 arc-sec. In order to avoid these errors, the invariant approach for the satellite gravity gradiometry (SGG) analysis has been proposed^12–14^. A significant progress has been made by the use of the perturbation theory for formulating the linearized model, which converts the non-linear least-squares minimization problem to a linear one with an acceptable computational costs, i.e. the additional computational effort per iteration reduces to reference gravity gradients synthesis^15^. Recently, the researchers performs the linearization for the second invariant $I_2$ from the Taylor expansion and obtained a gravity field model (IGGT_R1) from GOCE observations^16^.

Because the nonlinear effects of the third invariant $I_3$ are more complicated than that of the second invariant $I_2$, the existing results of gravity field determination from invariant are mostly based on $I_2$. However, it is found that $I_3$ has an advantage over $I_2$ in the effect of measurement noise under the condition that the measurement noise of gravitational gradient tensor have different levels like the GOCE mission (see more details in the “Results” and “Methods” sections). On the other hand, it is possible to provide a viable alternative tool and viewpoint for studies on satellite gravity gradients from the third invariant $I_3$. For these reasons, we recover a novel gravity field model named I3GG from the third invariant $I_3$ of the GOCE gravity gradient tensor and present the analysis of its characteristics by comparing with other models.

Because the third invariant $I_3$ is the sum of five products of three gravitational tensor coefficient matrix elements and the second one is only the sum of the six products of two gravitational tensor, the linearization of $I_3$ is more difficult than that of $I_2$^15^. In order to overcome this problem, this study employs the theory of boundary value problems with sphere approximation^11,17^, which formulates the relation between the invariant and the second-order radial derivatives of the gravitational potential with a relative accuracy of $10^{-9}$. To resolve this boundary value problem, we apply the 2D FFT method to derive a new gravity field model from the third invariant. Generally, there are two major steps to implement the 2D FFT method^18^: (1) obtaining the 2D Fourier spectrum and (2) transforming them into spherical harmonic coefficients. For the sake of the more efficient performance, the method we presented in this study makes modifications to both steps. In the first step, we use the coherent sampling theory instead of spherical harmonic integral discretization to obtain the relationship between the 2D FFT and the third invariant measurements. This sampling theory is applied to avoid the picket and spectral leakage effects, which leads to an explicit expression between spectrum values and the spatial signal. In the second step, we transform the 2D Fourier coefficients to spherical harmonic ones by using the pseudo-inverse via QR factorization, rather than computing the transformation integrals by recursions or numerical integrations. This contribution presents a new processing strategy to obtain a novel global gravity field model from the third invariant of the GOCE gravity gradient tensor based on a modified 2D FFT method, which can be regarded as an alternative approach to analysis the GOCE data analysis.

## Results

The gravity field determination from the third invariant depends on two kinds of data, i.e. the GOCE gravity gradient measurements and the synthetic gravity gradients derived from a priori gravity field. The GOCE gravity gradient measurements of the components $V_{xx}$, $V_{yy}$, $V_{zz}$ and $V_{xz}$ in the GRF are taken from the Level-2 product EGG_NOM_2 (ESA) from November 1, 2009 to August 1, 2012. The synthetic gravity gradients of the components $V_{xy}$ and $V_{yz}$ are derived from the global gravity field model EIGEN-5C. Then a new gravity field model (I3GG) is obtained by applying the theory of boundary value problems for the third invariant with sphere approximation and the modified 2D FFT method proposed based on the coherent sampling theory and the pseudo-inverse via QR factorization. Details are given in the “Methods” section.

**Figure 1:**
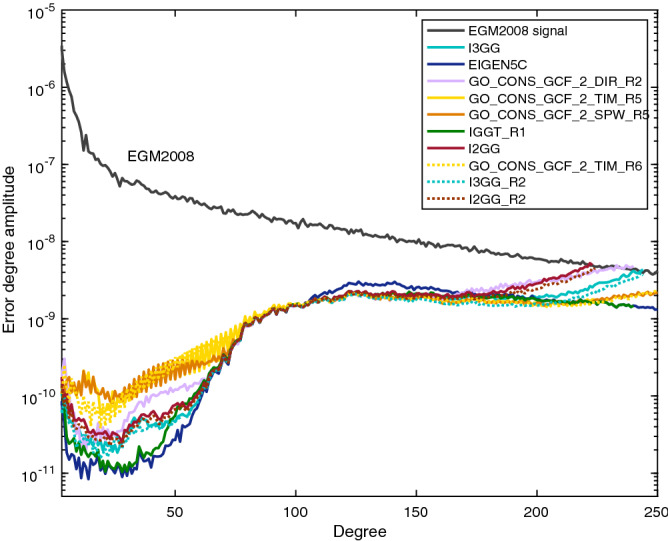
Difference degree amplitudes of the gravity field models DIR_R2, TIM_R5, SPW_R5, EIGEN-5C, IGGT_R1, I3GG, I2GG, TIM_R6, I3GG_R6, I2GG_R6 compared to EGM2008.

In order to evaluate its performance, the EGM2008 is taken as the reference and then we compare the error degree amplitudes between I3GG and other models, including the widely-used GOCE models (i.e. GO_CONS_GCF_2_DIR_R2 (DIR_R2), GO_CONS_GCF_2_TIM_R5 (TIM_R5) and GO_CONS_GCF_2_SPW_R5 (SPW_R5)), EIGEN-5C and IGGT_R1, as shown in Fig. 1. It should be pointed out that models DIR_R2, TIM_R5, SPW_R5 and IGGT_R1 are developed only from GOCE measurements and the regularization is applied to the coefficients of TIM_R5, SPW_R5 and IGGT_R1 at degrees above 200 while DIR is recovered without regularization^16,19^, which causes a larger maximum degree of recovery in the spectrum. From Fig. 1, it is seen that the maximum recovery degree of I3GG is 240, corresponding to a spatial resolution of 83 km at the equator. Since the signals of the recovered gravity model below the bandwidth of the band-pass filter mainly come from the reference model, results show that the I3GG and EIGEN-5C are relatively consistent at degrees below 27 ($\approx$ 5 mHz). The degree amplitudes of GOCE-derived models become higher than EIGEN-5C in the transition band (degrees 28–69). For degrees (70–200), all models except EIGEN-5C are very close to each other because that the signals in this bandwidth are mainly provided by GOCE gravity gradients measurements. The bumps of these curves are related to the improvements from GOCE mission to the inclusion of low-accuracy terrestrial data in certain regions in the EGM2008 model^4^. The amplitudes of the EIGEN-5C model are slightly higher in this bandwidth due to the contribution of GRACE data rather than GOCE data. Briefly, thanks to the advantages of the gradiometer measurements, the accuracy of the GOCE models adopted in this paper outperforms the EGM2008 and EIGEN-5C models in this spectral range. Between the models without regularization, the model I3GG is lower than the DIR_R2 for higher degrees (> 200). But in the same range of degrees, both of them are higher than the models which benefit from the regularization, i.e. the TIM_R5, SPW_R5 and IGGT_R1, or from the terrestrial gravity field data i.e. the EIGEN-5C.

**Figure 2:**
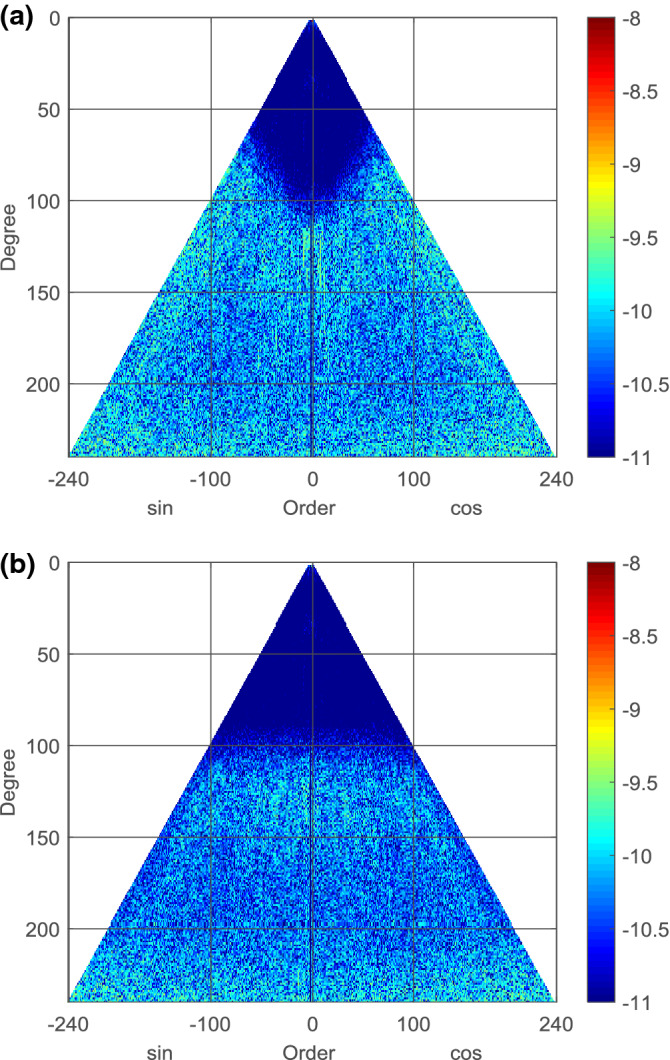
Spherical harmonic coefficients differences between I3GG and the existing gravity field models: (**a**) EGM2008 (**b**) EIGEN-5C, provide as absolute values in logarithmic scale ($\mathrm {log}_{10}$).

Figure 2 shows the I3GG coefficients deviation to EGM2008 and EIGEN-5C in logarithmic scale. The spherical harmonic coefficients of I3GG and EGM2008 shown in Fig. 2a fits well to each other for degree/order 0 to 70 and the coefficients deviation becomes larger for higher degrees/orders, which means that GOCE gravity field models contribute new information in high-frequency parts. The similar situation happens between the coefficients of I3GG and EIGEN-5C except for the fitted range that is up to degrees/orders 100, as shown in Fig. 2b. It can be also concluded that spherical harmonic coefficients of EIGEN-5C are more accurate than that of EGM2008 at degrees between 70 and 100, if we use the I3GG as a standard.

**Figure 3:**
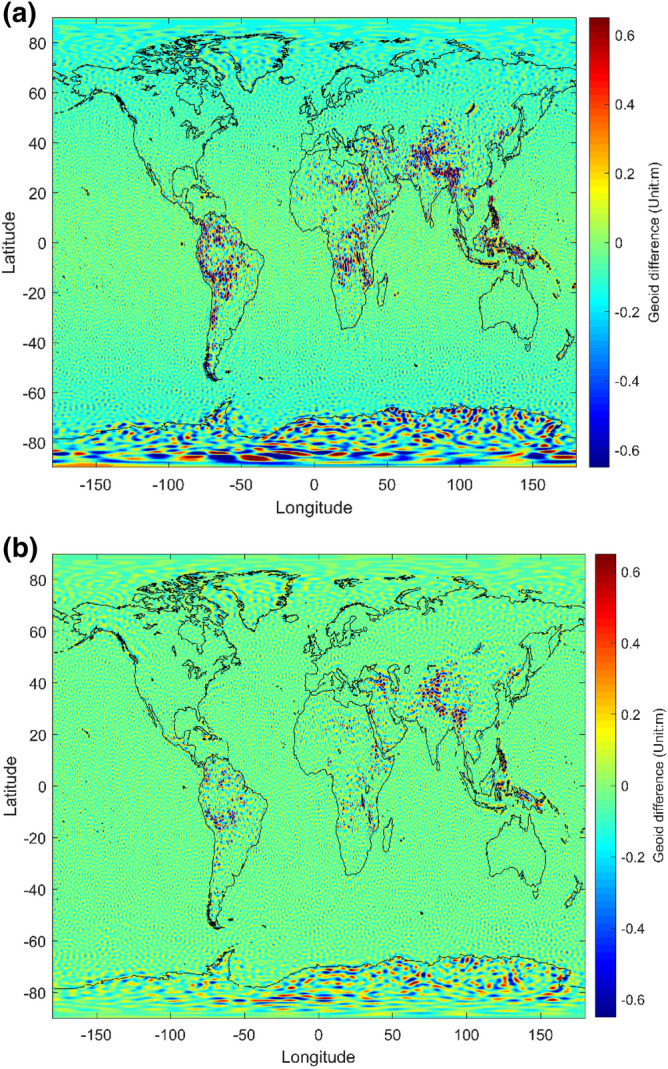
Cumulative geoid difference between I3GG and the existing gravity field models up to degree/order 210 (Unit: m): (**a**) EGM2008 (**b**) EIGEN-5C. These figures are generated by using Matlab(R2018a).

In addition to the spectral comparisons displayed in Figs. 1 and 2, the differences between these models are investigated in the spatial domain. First, we computed the cumulative geoid height deviation between the I3GG and EGM2008 (Fig. 3a) and EIGEN-5C (Fig. 3b) up to degree/order 210. From Fig. 3, it is obvious that at regions where no or poor quality terrestrial data are available (Himalaya, Africa, Amazonas and Antarctica) new gravity field information is added, which is due to the advantages of the gravity gradient measurements of the GOCE mission in the medium and short wavelength ranges.

**Figure 4:**
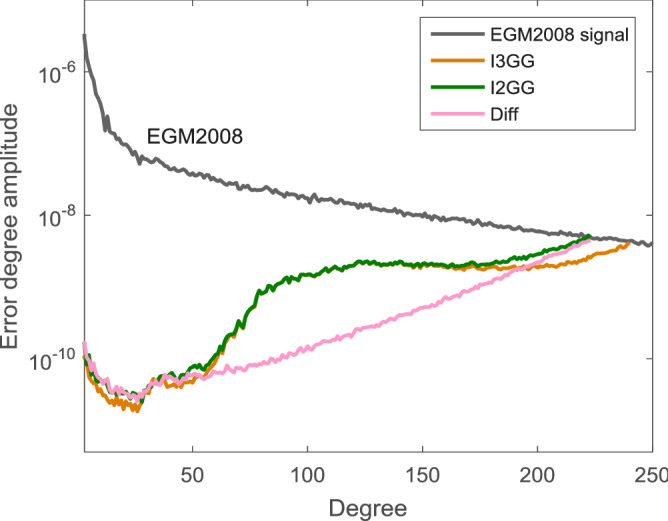
Difference degree amplitudes of the gravity field models I3GG and I2GG compared to the EGM2008.

Then we compute the RMS of the differences between these models from $1^\circ \times 1^\circ$ geoid height grids between $-80^\circ$ and $80^\circ$ latitude (i.e. without the polar caps). The RMS values are computed for the common whole-frequency range (degrees 0–210) and for the medium-frequency range (degree 70–150), where these models have a similar behaviour, as given in Table 1. Moreover, in order to inspect the differences between the invariant $I_2$ and $I_3$ in the gravity field determination, the same processing strategy is applied on the second invariant $I_2$ measurements of GOCE mission and we obtained another gravity field model named I2GG. Its RMS values of the geoid differences between other models are also given in Table 1 and accordingly its difference degree amplitudes from the I3GG are shown in Fig. 4.

**Table 1:** RMS values of geoid differences (unit: m) between in the eight models (I3GG, DIR_R2, TIM_R5, SPW_R5, I2GG, IGGT_R1, EIGEN-5C and EGM2008) for the common degree range of the models, i.e. 0–210 (lower triangle) and for the medium- frequency range 70–150 (upper triangle). The RMS values are computed from $1^\circ \times 1^\circ$ grids excluding the polar caps, i.e. latitude range of $-80^\circ$ and $80^\circ$.

	Degree range: 70–150							
	I3GG	DIR_R2	TIM_R5	SPW_R5	I2GG	IGGT_R1	EIGEN-5C	EGM2008
I3GG	–	0.072	0.072	0.071	0.014	0.055	0.059	0.090
DIR_R2	0.137	–	0.020	0.019	0.072	0.051	0.117	0.081
TIM_R5	0.119	0.083	–	0.011	0.074	0.052	0.118	0.081
SPW_R5	0.119	0.084	0.018	–	0.073	0.052	0.118	0.080
I2GG	0.076	0.145	0.101	0.139	–	0.054	0.060	0.091
IGGT_R1	0.091	0.117	0.128	0.101	0.107	–	0.101	0.087
EIGEN-5C	0.088	0.186	0.170	0.169	0.114	0.130	–	0.120
EGM2008	0.131	0.141	0.117	0.116	0.150	0.125	0.159	–
	Degree range: 0–210							

For the common whole-frequency range (0–210), Table 1 shows that the I3GG solution is closer to the invariant solutions I2GG and the IGGT_R1 (RMS = 0.076–0.091 m) than to the other GOCE models DIR_R2, TIM_R5, SPW_R5 (RMS = 0.119–0.137 m). In this case, the I3GG and I2GG solutions show a stronger consistency with the EIGEN-5C than the other GOCE models. When replacing the standard model by the EGM2008, the I3GG and IGGT_R1 solutions have lower RMS values of geoid differences than the ones except the regularized, i.e. the TIM_R5 and SPW_R5. For the medium-frequency range (70–150) it is visible that the difference among GOCE modes is much smaller (RMS $=$ 0.014–0.078 m) than the EGM2008 (RMS $=$ 0.081–0.120 m). Since I3GG, I2GG and IGGT_R1 have taken the EIGEN-5C as a priori model, they are closer to the EIGEN-5C than the DIR_R2, TIM_R5 and SPW_R5, which holds true both for the medium-frequency and the common whole-frequency range.

Meanwhile, the re-calibrated GOCE data released in 2018 is also dealt with and the corresponding gravity field model named I3GG_R2 and I2GG_R2 from the invariant $I_2$ and $I_3$, respectively, as shown in Fig. 1. It indicates that the I3GG_R2 and I2GG_R2 also agree well with other models for degree 70 to 150, which is the most sensitive bandwidth for the signals detected by GOCE mission. For lower and higher degrees they are slightly lower than I3GG and I2GG, respectively. The same phenomenon appears in the comparison between TIM_R5 and TIM_R6 (GO_CONS_GCF_2_TIM_R6). It should be pointed out that the Kaula-regularization applied to coefficients of degrees/orders 201–300 of both TIM_R5 and TIM_R6 so that they are almost the same in the range. Table 2 shows the RMS valus of geoid differences of TIM_R5 versus TIM_R6, I2GG versus I2GG_R2 and I3GG versus I3GG_R2 are 0.011, 0.008 and 0.039 for the medium-frequency range,and 0.038, 0.025 and 0.082 for the whole-frequency range, respectively. It indicates the re-calibration leads more improvements to the lower and higher degrees than the medium-frequency range. Additionally, if TIM_R6 is used as a standard, Table 2 shows that for the lower and higher degrees the rms values of geoid differences of the models from the invariant $I_3$, i.e. the I3GG and I3GG_R2, are lower than the ones from $I_2$, i.e. I2GG and I2GG_R2, respectively. Certainly, because that I2GG_R2 and I3GG_R2 are also derived from the re-calibrated GOCE data, their the rms values of geoid differences with TIM_R6 are lower than the ones of I2GG and I3GG, respectively.

Under the condition that the linearization error is negligible, the measurement noise has the main influence on the gravity field determination. As mentioned in the “Methods” section, the theoretical computation indicates that the effect of measurement noise on $I_3$ is lower than that on $I_2$ for GOCE mission, which also holds true for the realistic data. In order to avoid the deviations from methodology and data processing, the comparison between the I3GG and I2GG is investigated in this section. From Table 1, it is shown that the difference RMS values of I3GG are smaller than that of I2GG when compared to the EGM2008 or EIGEN-5C. Specifically, for the common whole-frequency range (0–210), the ratio of the difference RMS values of the I3GG to I2GG is 87.3% (0.131/0.150) when the EGM2008 is used as the reference, while the ratio is 77.2% (0.088/0.114) when EIGEN-5C is used. The two ratios are bounced around the theoretical value 81.6% [$\sqrt{4/3}/\sqrt{2}$ from Eq. (14)], and their relative deviation to the theoretical value are about 7% and 5%, respectively. From Fig. 4, it is also illustrated that the maximum degree of I2GG is 220, which is lower than that of I3GG (degree 240). However, they are close to each other in the medium-frequency range, which is the signal sensitive band of GOCE mission and has the highest signal to noise ratio. The same rerult can be obtained by the comparison between I3GG_R2 and I2GG_R2. Such comparisons above show that both the invariant $I_2$ and $I_3$ can recover the gravity field effectively from the GOCE measurements under the sphere approximation. Additionally, compared to the linearization error, the comparison indicates that the measurement noise is the major factor which, affects the accuracy and the resolution of the gravity field determination.

**Table 2:** RMS values of geoid differences (unit: m) of I2GG versus I2GG_R2, I3GG versus I3GG_R2, TIM_R5 versus TIM_R6, I2GG versus TIM_R6, I2GG_R2 versus TIM_R6, I3GG versus TIM_R6 and I3GG_R2 versus TIM_R6 for the common degree range of the models, i.e. 0–210 and for the medium- frequency range 70–150 . The RMS values are computed from $1^\circ \times 1^\circ$ grids excluding the polar caps, i.e. latitude range of $-80^\circ$ and $80^\circ$.

	Degree range: 70–150	Degree range: 0–210
I2GG versus I2GG_R2	0.008	0.025
I3GG versus I3GG_R2	0.039	0.082
TIM_R5 versus TIM_R6	0.011	0.038
I2GG versus TIM_R6	0.073	0.157
I2GG_R2 versus TIM_R6	0.067	0.136
I3GG versus TIM_R6	0.072	0.123
I3GG_R2 versus TIM_R6	0.044	0.076

An independent comparison with external data is made using geoid heights determined by GNSS positioning and leveling (GNSS/leveling). The details of the data processing procedures can be found in the study^20–22^. In order to verify and validate the method for the invariants, the models from the same GOCE data without re-calibration are compared, i.e. DIR_R2, TIM_R5, SPW_R5, and IGGT_R1 are used. Only the coefficients up to degree/order (d/o) 210 were used in this comparison. Considering the spatial resolution and the transition region effect, a Gaussian filter with a filter width of 47 km was applied to both the models and the GNSS/leveling data. Table 3 shows the results for the I3GG in comparison to other GOCE gravity field models using GNSS/leveling points of the European countries, including Norway, UK, Belgium, France, Germany and Netherlands. The RMS difference results show that I3GG has a relative comparative advantage over I2GG in Belgium and France. Meanwhile, in the rest countries they have alomst the same performance and their RMS differences are smaller than 1 cm . It should be pointed that the advantage of I3GG in the higher degrees is suppressed by the low-pass filter. Specifically, I3GG and I2GG fits better in comparison to the other models in UK. We also see that I3GG has an advantage over another second-invariant-derived model IGGT_R1 in all countries except Netherlands even though the IGGT_R1 applied regularization with the a-priori model EIGEN-5C. I3GG performs better in Norway, UK, Belgium, France and Germany of 0.3, 2.1, 2.9, 0.4 and 0.1 cm when compared with IGGT_R1. When compared with other GOCE-derived models DIR_R2, TIM_R5, SPW_R5, I3GG is in the middle level of the list in these countries. Briefly, it shows that the medium- to short-wavelength parts of the Earth’s gravity field can be obtained effectively by using $I_3$ from GOCE.

**Table 3:** The RMS difference (unit: m) between GNSS/leveling data in Norway, UK, Belgium, France, Germany and Netherlands and the model-derived geoid heights on the basis of EIGEN-5C and the GOCE-derived models I3GG, DIR_R2, TIM_R5, SPW_R5, I2GG, IGGT_R1 (number of points in brackets, for maximum d/o 210).

	Norway	UK	Belgium	France	Germany	Netherlands
	(− 1174)	(− 181)	(− 2707)	(− 1548)	(− 226)	(− 543)
I3GG	0.186	0.414	0.081	0.165	0.101	0.096
DIR_R2	0.183	0.440	0.123	0.162	0.120	0.121
TIM_R5	0.193	0.438	0.062	0.163	0.096	0.080
SPW_R5	0.193	0.443	0.072	0.165	0.097	0.093
I2GG	0.184	0.419	0.152	0.184	0.101	0.089
IGGT_R1	0.189	0.435	0.110	0.169	0.102	0.091
EIGEN-5C	0.192	0.437	0.062	0.169	0.089	0.090

## Discussion

This proposal for the gravity field recovery from the invariant in satellite gradiometry is to avoid the errors in the reconstitution procedure of the satellite’s attitude. In this paper, it is found that the third invariant $I_3$ is more competitive than $I_2$ on the gravity field determination GOCE mission, although $I_2$ is easier to be dealt with and get more attention from researchers. Accordingly, we use the third invariant $I_3$ of the GOCE gravity gradient tensor to obtain the I3GG. The theory of boundary value problems is adopted to implement linearization of the third invariant gravity gradients with an accuracy of $O(J_2^2\cdot T_{ij})$. In order to solve the boundary value problems efficiently, we propose an alternative method of 2D FFT, which uses the coherent sampling theory to obtain the relationship between the 2D FFT and the third invariant $I_3$ measurements and applies the pseudo-inverse via QR factorization to transform the 2D Fourier coefficients to spherical harmonic ones. The complexity of this algorithm reaches a level of $O(l_{max}^4)$ without the precomputation of the integrals by recursions or numerical integrations. Based on the theoretical bases mentioned above, the GOCE gravity gradient data of the nominal mission phase and the lower degree information from EIGEN-5C were combined in the construction of a satellite-only gravity field model to a maximum degree of 240, corresponding to a spatial resolution of 83 km at the equator. The results indicate that the I3GG agrees well with the other GOCE gravity field models in the medium-frequencies range (degree 70–150), which is the signal sensitive band of the GOCE mission. For the lower frequencies range, I3GG is close to the EGM2008 and EIGEN-5C and their fitted ranges are up to degree/order 70 and 100, respectively. From the point of view of the spatial domain, at regions where no or poor quality terrestrial data are available (Himalaya, Africa, Amazonas and Antarctica) new gravity field information is added. Moreover, for comparison purposes, we applied the same processing strategy on the second invariant $I_2$ measurements of the GOCE mission and obtained I2GG with a maximum degree of 220, which is 20 degrees lower than that of I3GG. Their RMS values of the geoid differences indicate that the effects of measurement noise of I3GG is about 20% lower than that of the I2GG model when compared to the EGM2008 or EIGEN-5C. The same characteristics can be also concluded when we dealt with the re-calibrated GOCE data. Briefly, the results show that the approach presented in this study is an effective way to obtain the gravity field models with high accuracy and spatial resolution from the third invariant of gravity gradient tensor. It is believed that it provides a viable alternative tool and viewpoint for studies on satellite gravity gradients.

## Methods

There are two basic theoretical aspects for the gravity field determination from $I_3$: the first is to formulate the relationship between the gravity potential coefficients and $I_3$, and the second aspect is the 2D FFT for dealing with the boundary value problems. The former relates to the linearization of invariant and its error level, while the latter relates to the data processing flow and efficiency, which will be discussed in the following two subsections, respectively.

### Invariant theory and the related boundary value problems

In this subsection the theory of the gravitational gradient tensor’s invariant and the related boundary conditions is introduced. The Earth’s gravity potential *V* satisfies the Laplace equation and leads the gravitational gradient tensor to the symmetric and trace-free, and therefore the following representation for invariant system $I_1$, $I_2$, $I_3$ is adopted^15^:

1a $$\begin{aligned} I_1&=V_{11}+V_{22}+V_{33}=0 \end{aligned}$$

1b $$\begin{aligned} I_2&=-\frac{1}{2}(V_{11}^2+V_{22}^2+V_{33}^2)-V_{12}^2-V_{13}^2-V_{23}^2 \end{aligned}$$

1c $$\begin{aligned} I_3&=V_{11}V_{22}V_{33}+2V_{12}V_{13}V_{23}-V_{11}V_{23}^2-V_{22}V_{13}^2-V_{33}V_{12}^2 \end{aligned}$$

Both $I_2$ and $I_3$, rather than $I_1$, are generally considered to be suitable to recover the gravity field models since that the first invariant is the trace of the gravitational tensor and its value is zero. In the next step, the invariant are linearized by the perturbations calculation relative to a priori known reference solution subject to

2 $$\begin{aligned} \delta {I_2}= & {} {I_2} - I_2^{ref}\nonumber \\= & {} - {U_{11}}{T_{11}} - {U_{22}}{T_{22}} - {U_{33}}{T_{33}}- 2\left( {{U_{12}}{T_{12}} + {U_{23}}{T_{23}} + {U_{13}}{T_{13}}} \right) + O\left( {T_{ij}^2} \right) , \end{aligned}$$

3 $$\begin{aligned} \delta {I_3}= & {} {I_3} - I_3^{ref}\nonumber \\= & {} \left( {U_{23}^2 - {U_{22}}{U_{33}}} \right) {T_{11}} + \left( {U_{13}^2 - {U_{11}}{U_{33}}} \right) {T_{22}}\nonumber \\&+ \left( {U_{12}^2 - {U_{11}}{U_{22}}} \right) {T_{33}} + 2\left( {{U_{12}}{U_{33}} - {U_{23}}{U_{13}}} \right) {T_{12}}\nonumber \\&+2\left( {{U_{23}}{U_{11}} - {U_{12}}{U_{13}}} \right) {T_{23}} + 2\left( {{U_{13}}{U_{22}} - {U_{12}}{U_{23}}} \right) {T_{13}} + O\left( {T_{ij}^3} \right) , \end{aligned}$$

where $I_2^{ref}$ and $I_3^{ref}$ are the priori invariant from reference gravity gradients $U_{ij}$, and $O(T_{ij}^2)$ and $O(T_{ij}^3)$ indicate the influences of terms which are non-linear in invariants. The disturbance gravity gradients $T_{ij}$ are defined as $T_{ij} = V_{ij} - U_{ij}$. Eqs. (2) and (3) are the linearized equations for invariant with an accuracy of $O(T_{ij}^2)$ and $O(T_{ij}^3)$, respectively^15^. Further researches focus on the simplicity of the linearization and reduces the computation to an efficient level with the sphere approximation^11,17^. The reference gravity potential *U* in the Eqs. (2) and (3) is approximated with ${\tilde{U}}=GM/r$ under the sphere approximation, and its second derivatives can be represented as

4 $$\begin{aligned} {{{{\tilde{U}}}}_{11}}= & {} {{{{\tilde{U}}}}_{22}} = - \frac{{GM}}{{{r^3}}},\nonumber \\ {{{{\tilde{U}}}}_{33}}= & {} \frac{{2GM}}{{{r^3}}},\nonumber \\ {{{{\tilde{U}}}}_{12}}= & {} {{{{\tilde{U}}}}_{13}} = {{{{\tilde{U}}}}_{23}} = 0. \end{aligned}$$

where *GM* is gravitational parameter of the Earth (*G* is the universal gravitational constant and *M* is the mass of the Earth). This approximation makes $\delta I_2$ and $\delta I_3$ with the accuracy of $O(J_2 \cdot T_{ij} )$ and $O(J_2^2 \cdot T_{ij} )$, respectively. Then one obtains the boundary value problems as follows^11^

5 $$\begin{aligned}&\left\{ {\begin{array}{*{20}{l}} {\Delta T = 0,}&{}{\mathrm{{on}\; \mathrm {or}\; \mathrm {out}\; \mathrm {of}\; \mathrm {the}\; \mathrm {sphere}\; \mathrm {surface}}}\\ {{T_{rr}}\left| {_s} \right. = - \frac{{{r^3}}}{{3GM}}\left( {\delta {I_2} + O\left( {{J_2} \cdot {T_{ij}}} \right) } \right) ,}&{}{\mathrm{{on}\; \mathrm {the}\; \mathrm {sphere}\; \mathrm {surface}}}\\ {T = O\left( {{r^{ - 1}}} \right) ,}&{}{r \rightarrow \infty } \end{array}} \right. \end{aligned}$$

6 $$\begin{aligned}&\left\{ {\begin{array}{*{20}{l}} {\Delta T = 0,}&{}{\mathrm{{on}\; \mathrm {or}\; \mathrm {out}\; \mathrm {of}\; \mathrm {the}\; \mathrm {sphere}\; \mathrm {surface}}}\\ {{T_{rr}}\left| {_s} \right. = - \frac{{{r^6}}}{{3{{\left( {GM} \right) }^2}}}\left( {\delta {I_3} + O\left( {J_2^2 \cdot {T_{ij}}} \right) } \right) ,}&{}{\mathrm{{on}\; \mathrm {the}\; \mathrm {sphere}\; \mathrm {surface}}}\\ {T = O\left( {{r^{ - 1}}} \right) ,}&{}{r \rightarrow \infty } \end{array}} \right. \end{aligned}$$

In this study the following second-order radial derivative for the Earth’s disturbance potential $T_{rr}$ is adopted^23^:

7 $$\begin{aligned} {T_{rr}}\left( {r,\theta ,\lambda } \right) = \frac{{GM}}{{{R^3}}}\sum \limits _{l = 2}^\infty {\left( {l + 1} \right) \left( {l + 2} \right) {{\left( {\frac{R}{r}} \right) }^{l + 3}}} \times \sum \limits _{m = 0}^l {\left( {{{{\bar{C}}}_{lm}}\cos m\lambda + {{{\bar{S}}}_{lm}}\sin m\lambda } \right) {{\bar{P}}_{lm}}\left( {\cos \theta \lambda } \right) } \end{aligned}$$

where $(r, \theta , \lambda )$ are the geocentric spherical coordinates (radius, co-latitude, longitude), *R* is reference radius. *l* and *m* are degree and order of spherical harmonic, ${{\bar{P}}}_{lm}\left( {\cos \theta \lambda }\right)$ are the fully normalized Legendre functions, ${{\bar{C}}}_{lm}$ and ${{\bar{S}}}_{lm}$ are fully normalized spherical harmonic coefficients. The ${\bar{C}}_{lm}$ and ${{\bar{S}}}_{lm}$ are the unknown parameters that should be estimated from observations as the solution of the gravity field recovery problem. By combining the Eqs. (6) and (7) we have access to the gravity field determination from the third invariant.

In addition, we compare the effects of measurement noises of the gravity gradients on the invariant $I_2$ and $I_3$ under the sphere approximation. The effects of measurement noise on them are also different since that different combinations of the invariant $I_2$ and $I_3$. Substituting Eq. (4) into (2) and (3) and ignoring the linearization errors $O(J_2 \cdot T_{ij} )$ and $O(J_2^2 \cdot T_{ij} )$, one obtains:

8 $$\begin{aligned} {T_{rr,\Delta {I_2}}}= & {} - \frac{1}{3}\left( {{T_{11}} + {T_{22}} - 2{T_{33}}} \right) \end{aligned}$$

9 $$\begin{aligned} {T_{rr,\Delta {I_3}}}= & {} - \frac{1}{3}\left( {2{T_{11}} + 2{T_{22}} - {T_{33}}} \right) \end{aligned}$$

where ${T_{rr,\Delta {I_2}}}$ and ${T_{rr,\Delta {I_3}}}$ are second-order radial derivatives derived from the invariants $I_2$ and $I_3$, respectively. Under the assumption that the measurement noises are uncorrelated^24^, error propagation then gives

10 $$\sigma _{{rr,\Delta I_{2} }} = \sqrt {\frac{{\sigma _{{11}}^{2} + \sigma _{{22}}^{2} + 4\sigma _{{33}}^{2} }}{9}}$$

11 $$\sigma _{{rr,\Delta I_{3} }} = \sqrt {\frac{{4\sigma _{{11}}^{2} + 4\sigma _{{22}}^{2} + \sigma _{{33}}^{2} }}{9}}$$

For the purpose of exploiting the influence of the measurement noises, here three situations are employed on the invariants:

1. When the noise levels of $T_{11}$, $T_{22}$ and $T_{33}$ are the same, it holds that $\sigma : = {\sigma _{11}} = {\sigma _{22}} = {\sigma _{33}}$, which results in
  12 $$\begin{aligned} {\sigma _{rr,\Delta {I_2}}} = \sqrt{\frac{2}{3}} \sigma ,\;\;{\sigma _{rr,\Delta {I_3}}} = \sigma \end{aligned}$$
2. When the noise levels of $T_{11}$ and $T_{22}$ are twice that of $T_{33}$, it holds that $\sigma : = \frac{1}{2}{\sigma _{11}} = \frac{1}{2}{\sigma _{22}} = {\sigma _{33}}$, which results in
  13 $$\begin{aligned} {\sigma _{rr,\Delta {I_2}}} = \sqrt{\frac{4}{3}} \sigma ,\;\;{\sigma _{rr,\Delta {I_3}}} = \sqrt{\frac{{11}}{3}} \sigma \end{aligned}$$
3. When the noise level of $T_{33}$ is twice that of $T_{11}$ and $T_{22}$, it holds that $\sigma : = {\sigma _{11}} = {\sigma _{22}} = \frac{1}{2}{\sigma _{33}}$, which results in
  14 $$\begin{aligned} {\sigma _{rr,\Delta {I_2}}} = \sqrt{2} \sigma ,\;\;{\sigma _{rr,\Delta {I_3}}} = \sqrt{\frac{4}{3}} \sigma \end{aligned}$$
  From the above results, it is concluded that when the noise level of $T_{33}$ are not worse than that of $T_{11}$ and $T_{22}$, the effect of measurement noise on $I_2$ is better than that on $I_3$. On the contrary, when the noise level of $T_{33}$ is higher than that of $T_{11}$ and $T_{22}$, the effect on $I_3$ is better than that on $I_2$. In reality, the measurement noises of GOCE mission corresponds to the third situation^2^, i.e. ${\sigma _{11}} = {\sigma _{22}} = \frac{1}{2}{\sigma _{33}} = 10\;\mathrm{{mE/H}}{\mathrm{{z}}^{1/2}}$, which makes $I_3$ more competitive than $I_2$ on the gravity field determination from this point of view. This issue has been discussed from the results of realistic data in the Results section.

### Principle of the alternative 2D FFT method

The 2D FFT method can be employed for the boundary value problems to determine the gravity field from the third invariant efficiently. As mentioned above, this method has two major steps, i.e. obtaining the 2D Fourier spectrum and transformation into spherical harmonic coefficients^18^. In this study an alternative 2D FFT method is developed to obtain the spherical harmonics from the third invariant in practice, which makes modifications to both steps.

First of all, we present a concise relation between the 2D FFT and the spatial signal based on the coherent sampling. The relation is developed from an explicit formula for reconstructing exactly a one-dimensional (1D) signal from the magnitude and phase of its FFT under the condition of coherent sampling, which is to avoid the picket fence and spectral leakage effects. In the case of a time series with a sampling rate of $f_s$ and *N* samples, while the value of the *k*-th sample of its 1D FFT is $(a+ib)$, the corresponding signal in time *t* domain $s_k(t)$ can be obtained as follows:

15 $$\begin{aligned} {s_k}\left( t \right) = {A_k}\cos \left( {2\pi {f_k}t + P{H_k}} \right) \end{aligned}$$

where ${f_k} = \left( {k - 1} \right) {f_s}/N$ is the frequency, $P{H_k} = \arctan \left( {b/a} \right)$ is the phase and ${A_k} = \frac{{\sqrt{{a^2} + {b^2}} }}{{Z\left( k \right) }}$ is the magnitude with

$$\begin{aligned} Z\left( k \right) = \left\{ {\begin{array}{*{20}{l}} N&{}{\mathrm{{for }}k = 1}\\ {\frac{N}{2}}&{}{{\mathrm{for}}\;2 \le k \le N} \end{array}} \right. \end{aligned}$$

It is noted that the first point ($k=1$) represents the 0 Hz frequecy. There is a similar formula for reconstructing a 2D signal from the magnitude and phase of its FFT. In the case of a spatial signal with a sampling rate of $f_{sx}$ and *N* sampling lines in x direction, and a sampling rate of $f_{sy}$ and *M* sampling lines in *y* direction, while the value located in the *h*-th row and *k*-th column of its 2D FFT is $(a+ib)$, the corresponding signal in space domain $s_{h,k}(x,y)$ can be obtained as follows:

16 $$\begin{aligned} {s_{h,k}}\left( {x,y} \right) = {A_h}\cos \left( {2\pi {f_h}x + P{H_h}} \right) \cdot {A_k}\cos \left( {2\pi {f_k}y + P{H_k}} \right) \end{aligned}$$

where ${f_x} = \left( {h - 1} \right) {f_{sx}}/N$ and ${f_y} = \left( {k - 1} \right) {f_{sy}}/M$ are the frequencies in *x* direction and *y* direction, respectively. The information of magnitude and phase are

17 $$\begin{aligned} P{H_h} + P{H_k} = \arctan \left( {b/a} \right) \end{aligned}$$

and

18 $$\begin{aligned} {A_h}{A_k} = \frac{{\sqrt{{a^2} + {b^2}} }}{{Z\left( {h,k} \right) }} \end{aligned}$$

respectively. The coefficient *Z*(*h*, *k*) is defined as

$$\begin{aligned} Z\left( {h,k} \right) = \left\{ {\begin{array}{*{20}{l}} {MN}&{}{\mathrm{{for }}\;h = 1\;\mathrm{{ and }}\;k = 1}\\ {\frac{{MN}}{2}}&{}{\mathrm{{for }}\;2 \le h \le M\;\mathrm{{ and }}\;k = 1,\;\mathrm{{or }}\;h = 1\;\mathrm{{ and }}\;2 \le k \le N}\\ {\frac{{MN}}{4}}&{}{\mathrm{{for}}\;2 \le h \le M\;\;\mathrm{{and }}\;2 \le k \le N} \end{array}} \right. \end{aligned}$$

Next, owing the fact that the Legendre functions can be expanded into sums of cosine or sine series, the same principles mentioned above can be also applied to discuss the correspondence between 2D FFT and spherical harmonics^25^. An analytical and square integrable function $f(\theta ,\lambda )$ defined on the unit sphere $(0\le \theta \le \pi , 0\le \lambda \le 2\pi )$ can be expanded in a series of spherical harmonics^26^

19 $$\begin{aligned} f\left( {\theta ,\lambda } \right) = \sum \limits _{l = 0}^{{l_{\max }}} {\sum \limits _{m = 0}^l {\left[ {{{{\bar{C}}}_{lm}}\cos \left( {m\lambda } \right) + {{{\bar{S}}}_{lm}}\sin \left( {m\lambda } \right) } \right] {{{\bar{P}}}_{lm}}\left( {\cos \theta } \right) } } \end{aligned}$$

For the sake of clarity, we first discuss the correspondence for a specific value of *l* and *m*:

20 $$\begin{aligned} {f_{lm}}\left( {\theta ,\lambda } \right) = \left[ {{{\bar{C}}_{lm}}\cos \left( {m\lambda } \right) + {{{\bar{S}}}_{lm}}\sin \left( {m\lambda } \right) } \right] {{\bar{P}}_{lm}}\left( {\cos \theta } \right) \end{aligned}$$

Using trigonometric product sum identities, we can obtain the coefficient $u_{lm}^q$ from the normalized Legendre function ${\bar{P}_{lm}}\left( {\cos \theta } \right)$ as follows^18^

21 $$\begin{aligned} {{\bar{P}}_{lm}}\left( {\cos \theta } \right) = \sum \limits _q^{} {u_{lm}^q\cos \left( {q\theta } \right) } \end{aligned}$$

for *m* is even, and

22 $$\begin{aligned} {{\bar{P}}_{lm}}\left( {\cos \theta } \right) = \sum \limits _q^{} {u_{lm}^q\sin \left( {q\theta } \right) } \end{aligned}$$

for *m* is odd, with

$$\begin{aligned} q = \left\{ {\begin{array}{*{20}{c}} {0,2,4,...l}&{}{\mathrm{{for }}\;l\;\mathrm{{ even}}}\\ {1,3,5,...l}&{}{\mathrm{{for }}\;l\;\mathrm{{ odd}}} \end{array}} \right. \end{aligned}$$

It is shown that the phase is zero in the latitude direction and the magnitude $u_{lm}^q$ can be obtained by a prior computation from the decomposition of the Legendre functions. In the case of spherical harmonics with a sampling rate of $f_{s\theta }$ and *N* sampling lines in $\theta$ direction, and a sampling rate of $f_{s\lambda }$ and *M* sampling lines in $\lambda$ direction, while the value located in the *h*-th row and *k*-th column of its 2D FFT is $(a+ib)$, the Fourier coefficients ${\Omega _{qm}}$ and ${\Delta _{qm}}$ are obtained as follows:

23 $$\begin{aligned} {\Omega _{qm}} = \frac{{\sqrt{{a^2} + {b^2}} }}{{u_{lm}^qZ\left( {h,k} \right) }}\cos \left( {P{H_k}} \right) \nonumber \\ {\Delta _{qm}} =\frac{{\sqrt{{a^2} + {b^2}} }}{{u_{lm}^qZ\left( {h,k} \right) }}\sin \left( {P{H_k}} \right) \end{aligned}$$

with

$$\begin{aligned} P{H_k} = \left\{ {\begin{array}{*{20}{l}} {\arctan \left( {b/a} \right) }&{}{{\mathrm{for }}\;m\;{\mathrm{even}}}\\ { - \arctan \left( {b/a} \right) }&{}{{\mathrm{for }}\;m\;{\mathrm{odd}}} \end{array}} \right. \end{aligned}$$

The frequencies in the *h*-th row and *k*-th column are ${f_\theta } = \frac{q}{{2\pi }}$ and ${f_\lambda } = \frac{m}{{2\pi }}$, respectively. Considering that only one component with a specific value of *l* and *m* is involved, the spherical harmonic coefficients ${{{\bar{C}}}_{lm}}$ and ${{{\bar{S}}}_{lm}}$ of Eq. (20) can be directly obtained from ${\Omega _{qm}}$ and ${\Delta _{qm}}$

24 $$\begin{aligned} {{{\bar{C}}}_{lm}}= & {} {\Omega _{qm}}\nonumber \\ {{{\bar{S}}}_{lm}}= & {} {\Delta _{qm}} \end{aligned}$$

Eqs. (21) and (22) indicate that the coefficient products ${\Omega _{qm}}u_{lm}^q$ is essentially the magnitude of spatial signal $\cos \left( {m\lambda } \right) \cos \left( {q\theta } \right)$ or $\cos \left( {m\lambda } \right) \sin \left( {q\theta } \right)$, and ${\Delta _{qm}}u_{lm}^q$ is the one of spatial signal $\sin \left( {m\lambda } \right) \cos \left( {q\theta } \right)$ or $\sin \left( {m\lambda } \right) \sin \left( {q\theta } \right)$. If only a spherical harmonic signal with a specific value of *l* and *m* is sampled under the condition of coherent sampling, the spherical harmonic coefficients ${{{\bar{C}}}_{lm}}$ and ${{{\bar{S}}}_{lm}}$ are equal to ${\Omega _{qm}}$ and ${\Delta _{qm}}$, respectively. When sampling the signal contains multiple spherical harmonics in Eq. (19), the coefficients ${\Omega _{qm}}$ and ${\Delta _{qm}}$ may contain the joint information of Fourier spectrum of these components since the signal aliasing appears in the spherical harmonics with the same order.

Then, in order to transform the 2D FFT of the third invariant into spherical harmonic coefficients, we present an alternative algorithm based on the pseudo-inverse via QR factorization in this step. Using trigonometric product sum identities, we can first obtain the normalized Legendre function ${{\bar{P}}_{lm}}\left( {\cos \theta } \right)$ as follows^26^:

25 $$\begin{aligned} {{\bar{P}}_{lm}}\left( {\cos \theta } \right) = \sum \limits _{k = 0}^l {{h_{lmk}}\left\{ {\begin{array}{*{20}{c}} {\cos k\theta ,}&{}{\mathrm{{for }}\;m\;\mathrm{{even}}}\\ {\sin k\theta ,}&{}{\mathrm{{for }}\;m\;\mathrm{{odd}}} \end{array}} \right. } \end{aligned}$$

where $h_{lmk}$ is the decomposition factor of the associated Legendre functions. For each order *m* the decomposition factors can be defined as a factor matrix as follows:

26 $$\begin{aligned} \mathbf{{H}} = \left( {\begin{array}{*{20}{c}} {{h_{m,m,0}}}&{}{{h_{m + 1,m,0}}}&{} \cdots &{}{{h_{L,m,0}}}\\ {{h_{m,m,1}}}&{}{{h_{m + 1,m,1}}}&{} \cdots &{}{{h_{L,m,1}}}\\ \vdots &{} \vdots &{} \ddots &{} \vdots \\ {{h_{m,m,L}}}&{}{{h_{m + 1,m,L}}}&{} \cdots &{}{{h_{L,m,L}}} \end{array}} \right) \end{aligned}$$

Therefore, we can easily obtain the spherical harmonic coefficients ${{{\bar{C}}}_{lm}}$ and ${{{\bar{S}}}_{lm}}$ from the Fourier coefficients ${\Omega _{qm}}$ and ${\Delta _{qm}}$ by solving linear equations as follows:

27 $$\begin{aligned} {\mathbf{{C}}_{\mathrm{{coef}}}} = {\mathbf{{H}}^{ - 1}}{\mathbf{{D}}_{\mathrm{{FFT}}}} \end{aligned}$$

where ${\mathbf{{C}}_{\mathrm{{coef}}}}$ is the vector of spherical harmonic coefficients ${{{\bar{C}}}_{lm}}$ and ${{{\bar{S}}}_{lm}}$, ${\mathbf{{D}}_{\mathrm{{FFT}}}}$ is the vector of Fourier coefficients ${\Omega _{qm}}$ and ${\Delta _{qm}}$. Since $\mathbf {H}$ is left-invertible, its columns are linearly independent and the QR factorization $\mathbf{{H}} = \mathbf{{QR}}$ exists. We have

28 $$\begin{aligned} {\mathbf{{C}}_{\mathrm{{coef}}}} = {\mathbf{{R}}^{ - 1}}{\mathbf{{Q}}^T}{\mathbf{{D}}_{\mathrm{{FFT}}}} \end{aligned}$$

We can compute the pseudo-inverse using the QR factorization to obtain the spherical harmonic coefficients. By taking advantages of the orthogonality between the even degree Legendre functions and the odd degree ones in the same order, we can reduce the computation based on the classification by parity of their degrees. Considering Eq. (6), one can see that the third invariant is derived from $f(\theta ,\lambda )$ by adding a multiplication factor *F*(*l*) for a specific degree *l*, where

29 $$\begin{aligned} F\left( l \right) = - \frac{3}{{{r^6}}}\frac{{{{\left( {GM} \right) }^2}}}{{{R^3}}}(l + 1)(l + 2){\left( {\frac{R}{r}} \right) ^{l + 3}} \end{aligned}$$

Finally, it leads to an explicit expression for computing the gravity field from $I_3$ measurements based on the Fourier analysis of spherical harmonics as follows

30 $$\begin{aligned} {\mathbf{{C}}_{\mathrm{{coef}}}} = {\mathbf{{F}}^{ - 1}}{\mathbf{{R}}^{ - 1}}{\mathbf{{Q}}^T}{\mathbf{{D}}_{\mathrm{{FFT}}}} \end{aligned}$$

where $\mathbf {F}$ is a diagonal matrix constructed by multiplication factors *F*(*l*). The time complexity of the method presented in this study is mainly determined by the 2D-FFT computation and the pseudo-inverse via QR factorization. When the maximum degree is $l_{max}$, the time complexity of the 2D-FFT computation is $O(l_{max}^2\cdot log(2l_{max}))$. Meanwhile, the pseudo-inverse has a running time of $O(l_{max}^3)$ for each order, so that the total complexity for all orders is $O(l_{max}^4)$.

### Realistic data processing

**Figure 5:**
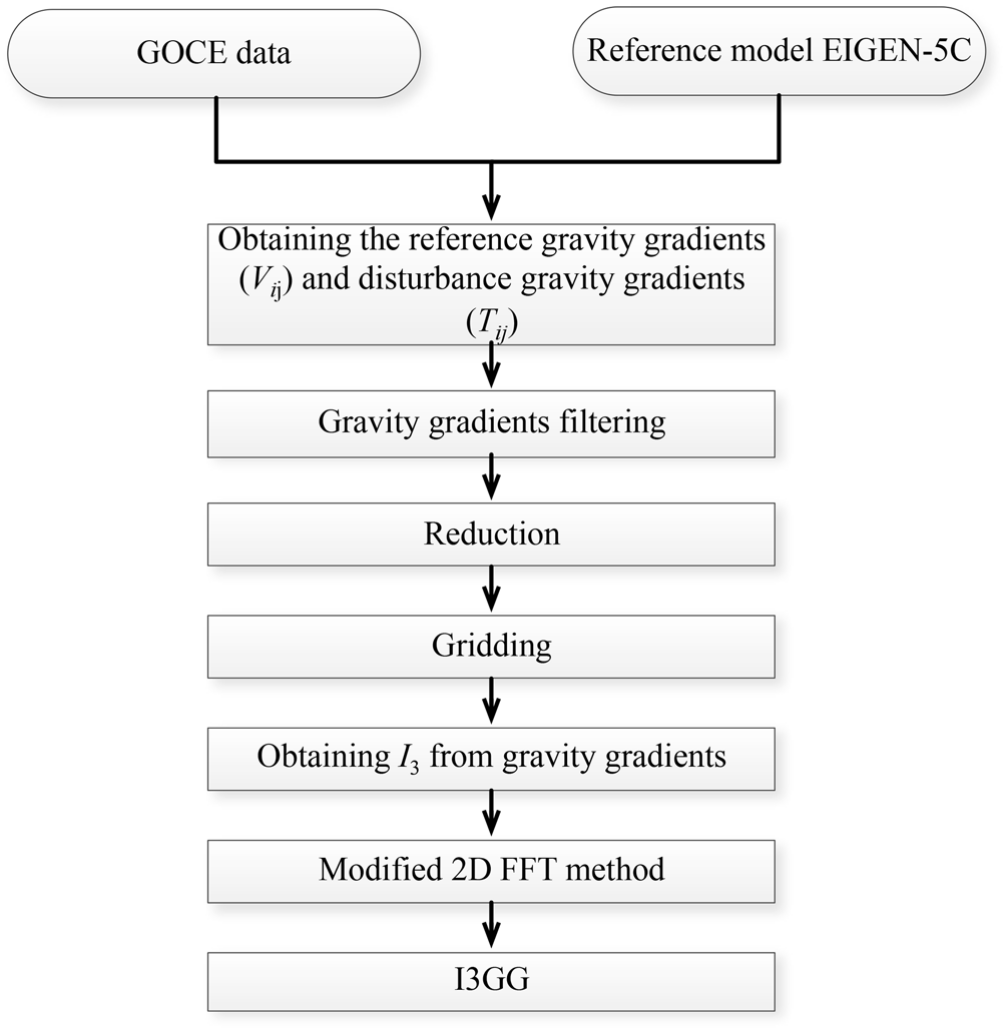
The flow chart of the gravity field determination from the third invariant by the 2D FFT method.

The components $V_{xx}$, $V_{yy}$, $V_{zz}$ and $V_{xz}$ are provided in the gradiometer reference frame (GRF) with highest accuracy of 10 to 20 mE. However, the accuracy of the other gravity gradients is reduced by around two orders of magnitude because they are measured by the less sensitive axes of the accelerometers^2^. Therefore, the gravity field determination from the third invariant depends on two kinds of data, i.e. the GOCE gravity gradient measurements and the synthetic gravity gradients derived from a priori gravity field. The GOCE gravity gradient measurements of the components $V_{xx}$, $V_{yy}$, $V_{zz}$ and $V_{xz}$ in the GRF are taken from the Level-2 product EGG_NOM_2 (ESA) from November 1, 2009 to August 1, 2012. The synthetic gravity gradients of the components $V_{xy}$ and $V_{yz}$ are derived from the global gravity field model EIGEN-5C. The synthetic gravity gradient values are first computed in the local north oriented frame (LNOR) and then transformed to the GRF by the attitude quaternions products. Due to the fact that the components $V_{xy}$ and $V_{yz}$ are very small compared to the main diagonal components, the accuracy of the synthetic gravity gradients satisfies the requirement for the gravity field determination from invariant^15^. On the other hand, the gravity field model EIGEN-5C is employed as the reference model and used to fill the polar gap since the inclination of the orbit of GOCE mission is $96.7^{\circ }$^8^. Meanwhile, the disturbance gravity gradients $T_{ij}$ can be also obtained by the subtraction of the reference gravity gradients $U_{ij}$ of EIGEN-5C from the GOCE gravity gradients measurements. Then the disturbance gravity gradients $T_{ij}$ are filtered to 5–100 mHz by a finite impulse response (FIR) filter, which means that the signals of the recovered gravity model at lower frequencies (i.e. below 5 mHz) mainly come from the reference model. The gravity gradients also need a vertical reduction from the realistic orbit height to the mean orbital sphere. Considering that the eccentricity of GOCE is about 0.001 and the difference between the realistic orbit height and the mean one is less than 4 km, according to Eq. (5), we can reduce the third invariant in the radial direction by using the Taylor series expansion

31 $$\begin{aligned} {\left. {\frac{{{\partial ^2}T}}{{\partial {r^2}}}} \right| _{{\tilde{S}}}} = {\left. {\frac{{{\partial ^2}T}}{{\partial {r^2}}}} \right| _S} + {\left. {\frac{{{\partial ^2}T}}{{\partial {r^3}}}} \right| _S} \cdot \delta r \end{aligned}$$

where $\delta r$ is the radial distances between the average sphere ${\tilde{S}}$ and realistic orbit *S*. The error caused by this procedure is less than 1 mE and meets the requirement of gravity field determination from GOCE mission^27^. Then, the measurements are gridded with a spacing of $0.5^{\circ } \times 0.5^{\circ }$ at the mean orbital sphere. For the sake of clarity, the associated flow chart is displayed in Fig. 5.

## Data Availability

The supplement related to this article is available online at: http://doi.org/10.5281/zenodo.2374753.
